# Movement seasonality in a desert-dwelling bat revealed by miniature GPS loggers

**DOI:** 10.1186/s40462-019-0170-8

**Published:** 2019-08-16

**Authors:** Irene Conenna, Adrià López-Baucells, Ricardo Rocha, Simon Ripperger, Mar Cabeza

**Affiliations:** 1 Global Change and Conservation Faculty of Biological and Environmental Sciences, University of Helsinki, PO Box 65, Viikinkaari 1, 00014 Helsinki, Finland; 2 Helsinki Institute of Sustainability Science, University of Helsinki, Helsinki, Finland; 3 Centre for Ecology Evolution and Environmental Changes (cE3c) Faculty of Sciences, University of Lisbon, 1749-016 Lisbon, Portugal; 4 Granollers Museum of Natural Sciences, 08402 Granollers, Catalonia Spain; 5 Conservation Science Group Department of Zoology, University of Cambridge, CB2 3EJ Cambridge, UK; 6 Museum für Naturkunde, Leibniz-Institute for Evolution and Biodiversity Science, Berlin, Germany; 7 Smithsonian Tropical Research Institute, Balboa, Ancón Panama

**Keywords:** Animal movement, Biologging, *Lavia frons*, GPS technology, Seasonal changes, Telemetry

## Abstract

**Background:**

Bats are among the most successful desert mammals. Yet, our understanding of their spatio-temporal dynamics in habitat use associated with the seasonal oscillation of resources is still limited. In this study, we have employed state-of-the-art lightweight GPS loggers to track the yellow-winged bat *Lavia frons* in a desert in northern Kenya to investigate how seasonality in a desert affects the a) spatial and b) temporal dimensions of movements in a low-mobility bat.

**Methods:**

Bats were tracked during April–May 2017 (rainy season) and January–February 2018 (dry season) using 1-g GPS loggers. Spatial and temporal dimensions of movements were quantified, respectively, as the home range and nightly activity patterns. We tested for differences between seasons to assess responses to seasonal drought. In addition, we quantified home range overlap between neighbouring individuals to investigate whether tracking data will be in accordance with previous reports on territoriality and social monogamy in *L. frons*.

**Results:**

We obtained data for 22 bats, 13 during the rainy and 9 during the dry season. Home ranges averaged 5.46 ± 11.04 ha and bats travelled a minimum distance of 99.69 ± 123.42 m/hour. During the dry season, home ranges were larger than in the rainy season, and bats exhibited high activity during most of the night. No apparent association with free water was identified during the dry season. The observed spatial organisation of home ranges supports previous observations that *L. frons* partitions the space into territories throughout the year.

**Conclusions:**

Our results suggest that, in low-mobility bats, a potential way to cope with seasonally harsh conditions and resource scarcity in deserts is to cover larger areas and increase time active, suggesting lower cost-efficiency of the foraging activity. Climate change may pose additional pressures on *L. frons* and other low-mobility species by further reducing food abundances.

**Electronic supplementary material:**

The online version of this article (10.1186/s40462-019-0170-8) contains supplementary material, which is available to authorized users.

## Background

Space use in animals is influenced by a combination of intrinsic factors, such as movement abilities and costs, and external factors, such as competition with other individuals/species and the availability and distribution of resources in the landscape [1, 2]. Seasonality, i.e. periodical fluctuations in environmental factors, is known to affect animal movements in a variety of ways by changing the abundance of resources in space and time [1, 3].

In hot deserts, which are typified by elevated temperatures and limitations in water sources, the seasonality of precipitation controls basic ecosystem processes and determines extreme fluctuations in resource availability [4]. Desert-dwelling species have developed a multitude of different strategies to cope with these challenging conditions [5–8]. However, current climate change projections estimate that deserts will face an increase in the frequency and duration of extreme droughts and reductions in water availability [9, 10], likely moving environmental conditions closer to the critical biological limits of desert animals [11].

Although attention directed to desert species in general is still low [11, 12], interest towards desert-dwelling bats has recently increased following concerns regarding their long-term persistence under climate change [13, 14]. Bats are considered a successful order of mammals in deserts thanks to their flight skills and, for desert specialist species, to urine concentration abilities that allow to limit water losses via excretion [5, 15–18]. Powered flight allows bats to efficiently track ephemeral resources and consequently buffers bats from some of the constraints imposed by deserts. For example, bats have been found to successfully use temporary water ponds available during wet periods, and to convey to permanent water bodies during dry seasons [19]. However, strategies to cope with prolonged drought may vary substantially across species, and important knowledge gaps still exist. Desert-dwelling bats’ sensitivity to climate change has been linked to exposure to high levels of evaporative water loss during day roosting and foraging [5, 20]. Additionally, they rely on a food resource, i.e. insects, which fluctuates greatly following rain patterns and is extremely limited during dry seasons or drought conditions [21–23].

While bats in temperate regions cope with a wintertime drop in food resources by undergoing periods of hibernation, seasonal thermic excursions are typically less pronounced in hot deserts and bats are active throughout the year, thus needing to employ various strategies to cope with seasonally harsh conditions.

Recent studies have identified a fundamental role of waterbodies in supporting diverse desert bat communities and their reproductive success [14, 19, 24, 25]. Permanent water pools have been shown to be particularly important during dry periods, as bats appear to converge around water points for a stable source of water and insects [19]. For example, Geluso and Geluso [26] found that capture rates at a permanent water pond in New Mexico were considerably higher during drought years, when no other water sources were available for bats in the area. On the other hand, permanent water bodies are rare in desert environments, and commuting daily over large distances to exploit these resources may be too costly or unfeasible for species with limited mobility skills, such as bats with low wing loading and aspect ratio [27]. It is unclear how relatively low-mobility bats cope with resource scarcity during dry seasons and whether they engage in long commuting flights or alternative strategies to mitigate for the potential consequences of seasonal decreases in resource availability.

In this study, we employed state-of-the-art GPS loggers to track the yellow-winged bat *Lavia frons* in a desert in northern Kenya with the aim of investigating how seasonality in a desert affects the a) spatial and b) temporal dimensions of movements in a low-mobility bat. To achieve this, we described home range and activity patterns during the dry and rainy seasons and tested for seasonal differences. We hypothesise that, compared to the rainy season, bat movements during the dry season will reflect an increased effort to locate resources. In particular, we predict that in the dry season, compared to the rainy one, a) home ranges will be larger to allow bats to potentially incorporate more resources, and b) activity patterns will be altered, with high activity sustained for a longer period to ensure sufficient food intake. Additionally, as the species has been observed to actively defend territories, but data on spacing patterns are limited, we quantified home range overlap between neighbours.

## Methods

### Study species and area

*Lavia frons* is one of five Megadermatidae bat species. It is distributed in Sub-Saharan Africa, reaching south to northern Zambia and Malawi, roughly between 15°N and 15°S [28]. It occupies savannah and semi-wooded areas, and only marginally extends to drier areas and deserts, where it is strongly connected to riparian habitats and to the presence of *Acacia* spp. [29]. The species is a low-mobility bat with low wing loading and aspect ratio [27], and a slow but highly agile flight, which allows for high manoeuvrability through dense, thorny vegetation. *L. frons* exhibit a hang-and-wait hunting strategy: they hang from a perch from which they scan the environment in search of prey by means of highly modulated pulses and by passive listening [28]. Observational studies conducted in the 1980s suggest that the species is monogamous and territorial, with members of a pair roosting nearby and sharing the same territory [28].

**Fig. 1:**
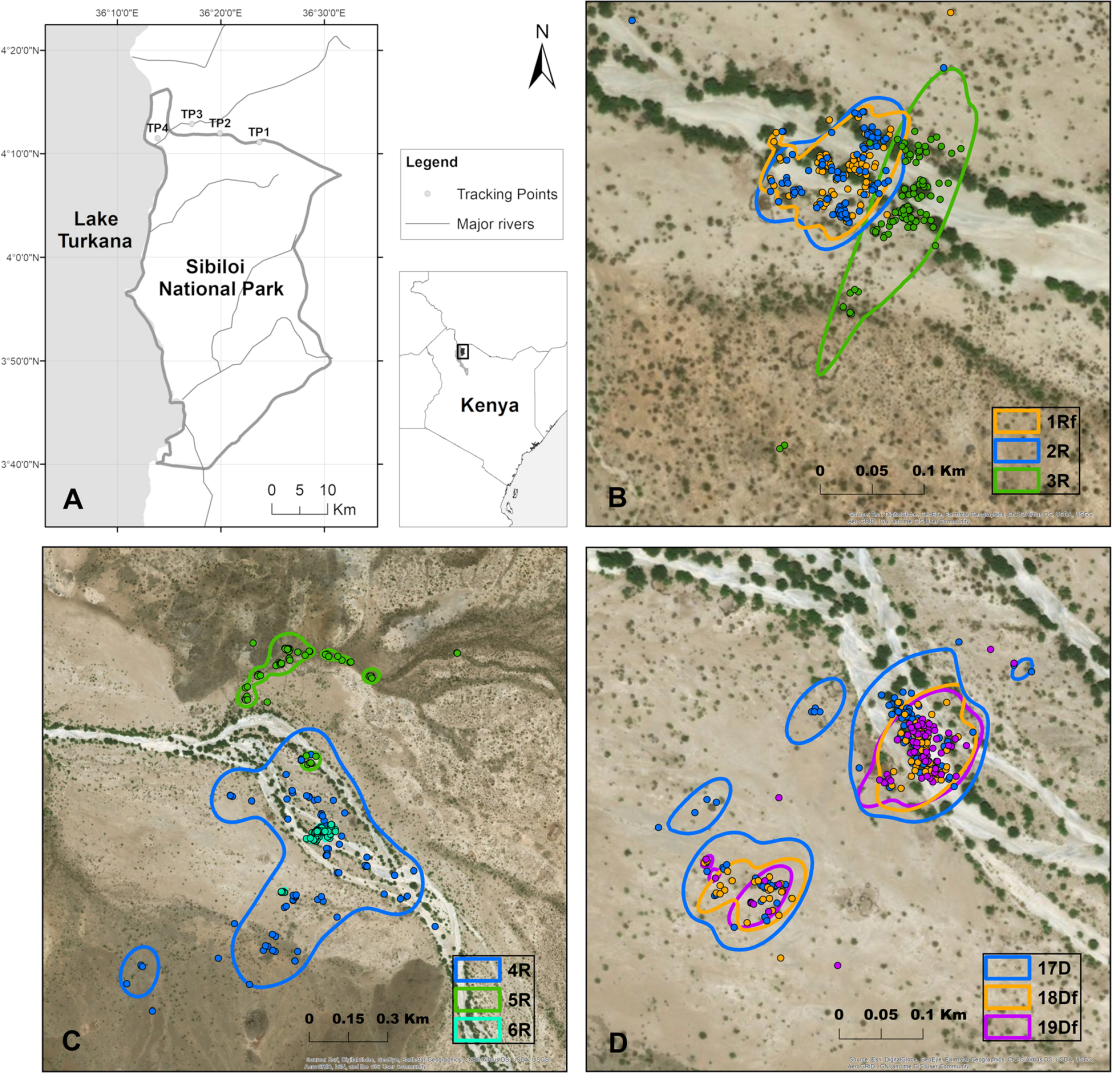
Panel **a** shows the study area and the tagging points (TP1 to TP4). Panels **b** to **d** show recorded locations (dots) and respective home range boundaries (lines) for individuals tracked in TP1 and TP2 during the rainy season, and TP2 during the dry season, respectively. Individuals are identified by different colours and IDs (see Table 1 for IDs’ list)

Our study was conducted along the north border of Sibiloi National Park (hereafter ‘Sibiloi NP’), Kenya (Fig. 1a). The park, which extends for an area of 1570 km^2^, is located along the shores of the world’s largest desert lake, Lake Turkana [30]. The climate in the area shows features of a desert due to its aridity [31]. The area has a solar insulation of 250 W/m^2^, an annual mean temperature of 32 °C and annual precipitation of ca. 130 mm [32], the latter showing great variations between years. Precipitation occurs during two rainy seasons, from November to December and March to May, which are separated by dry seasons, from January to February and June to October, the former also characterised by the highest yearly temperatures [33]. Except for the semi-saline Lake Turkana and ephemeral wells dug by local communities, no open water sources are available during the dry season. The vegetation is characterised by grassland or dwarf bushland (ca. 83%) and bushland (ca. 16%), while tree cover is supported only along ephemeral rivers (< 0.5%) [32]. These rivers, or ‘lagas’ (Additional file 1: Figure S1 and S2), are dry most of the year and retain water only during heavy rains, when flash floods occur. However, water persists underground, sustaining trees. Among these, *Salvadora persica* trees remain leaved throughout the year, thus providing shade during the hottest and dry periods and constituting the major roosting sites for *L. frons* in this area, while *Acacia* spp. are only marginally used (personal observation). Our study sites were located along a river, where the presence of *L. frons* had been confirmed during previous surveys.

### GPS tracking

Tracking sessions were conducted from April 5th to May 15th 2017 (rainy season) and from January 28th to March 1st 2018 (dry season). Humidity levels in the study area were monitored during the study period (EasyLog, EL-USB-2). Average daily humidity was 47.5% during the rainy season (31 days), 34.8% during the dry season (27 days) and 32.7% during the 30 days preceding the dry season. During each season, bat tracking was performed at four tagging points (TP1 to TP4, Fig. 1a) of increasing distance from the lake, to cover possible variability in environmental conditions linked to the presence of the lake (e.g. different humidity levels due to evaporation) and to minimise the influence of local landscape features in the home range estimates [21]. Tagging points coordinates are: 36.39485 E, 4.18532 N (TP1); 36.33191 E, 4.19983 N (TP2); 36.28669 E, 4.21442 N (TP3); 36.23121 E, 4.19171 N (TP4).

Bats were captured after dusk using mist nets and examined to determine sex, age, forearm length, weight and reproductive status. Tracking equipment (hereafter only “tag”) was then fitted on partially trimmed fur by applying a small quantity of cyanoacrylate-based glue, and was comprised of a rechargeable GPS logger (PinPoint 10, Biotrack Ltd., Dorset, UK) attached to a VHF transmitter (Biotrack or Telemetrie-Service Dessau or Holohil). The VHF was primarily intended to allow for tag recovery after dropping from the bat given the impossibility to remotely download the GPS data. The tag weighed 1.45 g (1 g GPS + 0.31–0.35 g VHF + glue weight), which represented between 6.1 and 9.1% of a bat’s weight (on average 18.36 g ± 2.29, see Table 1). These values are above the best practice threshold of 5% [34], but remain below the 10% threshold considered adequate for short-term studies [35, 36]. Additionally, clutter-space foragers, such as *L. frons*, characterised by broad wings and low wing loading, are expected to be able to carry larger loads than narrow-winged species, as they have the ability to manipulate wing shape to adapt flight [35]. Furthermore, recapturing some bats allowed us to verify that the weight of the bat had not changed over the short tracking period, and that skin was not injured by tag attachment (see Table 1, Additional file 1: Figure S3). All tracked bats were in a non-reproductive state, except for one female that showed a late lactation stage and was not carrying any dependent offspring. The GPS tags were set to record locations (hereafter “fixes”) for seven nights according to the following schedule: from 18:00 to 22:00 every 30 min, from 22:00 to 05:00 every 60 min, and one additional fix at 05:30. Bats from the same tagging point were tracked over the same period or, in a minority of cases, within a few days from each other. Tags remained attached to the bat for 4 to 14 days and were recovered either from the bat during recapture or, once the tag was shed, from the ground by radio-tracking the VHF signal. Radio-tracking was undertaken using a three-element Yagi antenna (rigid and flexible Yagi antennas, Biotrack) and a receiver (ICOM radio-receivers IC-R20 and Biotrack SIKA). The research was conducted under the following permits: National Commission for Science, Technology and Innovation (NACOSTI) n. NACOSTI/P/16/21446/14491 and NACOSTI/P/18/21446/20296. Animals were handled following guidelines listed by the Bat Conservation Trust [37].

**Table 1:** Tagging point, sex, weight, tracking parameters and values of home range and core area for each of the 22 individuals considered in the analyses. Weight are measured in g and areas in ha

Season	ID	Tagging point	Sex	Capture weight	Recovery weight	N. of fixes	N. of nights	% of tag weight	Core area	Home range
Rainy	1Rf	TP1	f	22.1		84	6	6.56	0.17	1.06
	2R	TP1	m	16.7		94	7	8.68	0.32	1.30
	3R	TP1	m	17.1		96	7	8.46	0.27	1.71
	4R	TP2	m	16.3		78	6	8.90	10.7	42.32
	5R	TP2	m	17.5		102	7	8.29	0.55	3.67
	6R	TP2	m	15.9	15.1	105	7	9.12	0.07	0.45
	7R	TP3	m	17.0		92	7	8.53	0.10	1.54
	8R	TP3	m	15.9		97	7	9.12	0.17	1.30
	9R	TP3	m	16.9		93	6	8.58	0.02	0.53
	10R	TP4	m	18.2		116	8	7.98	0.09	0.85
	11R	TP4	m	16.0		62	5	9.06	0.03	0.46
	12Rf	TP4	f	19.1		70	5	7.59	0.06	0.58
	13Rf	TP4	f	20.1		56	4	7.21	0.19	2.32
Dry	14D	TP1	m	17.2		99	7	8.44	0.13	0.92
	15D	TP1	m	16.4		64	5	8.83	0.16	2.06
	16Df	TP1	f	22.4	22.7	72	5	6.46	0.24	1.33
	17D	TP2	m	16.7	17.2	102	7	8.68	0.57	4.76
	18Df	TP2	f	20.1	19.7	87	6	7.20	0.26	1.95
	19Df	TP2	f	23.7	23.2	69	5	6.13	0.18	1.70
	20Df	TP3	f	20.2		76	6	7.17	1.40	36.96
	21D	TP4	m	17.4		61	5	8.31	0.51	10.45
	22Df	TP4	f	21.0		27	na	6.90	0.08	1.80

Out of 29 tagged bats, 13 tags were retrieved during the rainy season and 9 during the dry season, determining a final sample of 22 (75.9% of deployed tags). After removing failed and low-quality fixes (Horizontal Dilution of Precision ≥10) and fixes whose positions may have been affected by bat handling, a total of 1802 fixes were available for the analyses, where each bat averaged 81.9 ± 20.1 fixes spread over 6.1 ± 1.0 nights (a night counted when ≥5 fixes were successfully recorded, see Table 1).

### Data analysis

#### Influence of seasonality on the spatial dimension of movement: home range

Successive locations were not considered temporally autocorrelated (for definition of temporal autocorrelation see for example [38, 39]) as the minimum interval of 30 min employed was sufficient for *L. frons* to cross its home range, the average distance of the two furthest points recorded for each bat being 431.7 ± 329.3 m (considering 95% of all points). For comparison, a bat species with wing morphology similar to that of *L. frons*, *Plecotus auritus*, was found to cover 1 km in 5.4 min [27, 40]. Home ranges and core areas were estimated as the area included within the 95 and 50% contours, respectively, of fixed Kernel Density Estimation (hereafter just “kernel”). The contours were determined based on the smoothing parameter calculated via plug-in method following performance tests performed in Lichti and Swihart [41] (see Additional file 2 for further detail on home range estimator selection). Following the recommendation in Seaman et al. [42] for estimating home ranges via kernel when a representative sample of ≥30 is available, all bats with a minimum of 30 fixes were included in the analyses. To allow for sample size as large as possible, an additional individual approximating this threshold (ID 22Df, 27 fixes, see Table 1) was included in the home range size test (*n* = 22) after verifying that its home range was fully revealed. This was done by plotting the number of fixes against the area of the corresponding minimum convex polygons and verifying that an asymptote was reached. However, individual 22Df was excluded from analyses of home range overlap and activity patterns (*n* = 21). Calculations were performed using the R package “ks”, and parametrisation of the plug-in method was conducted following Duong [43]. Core areas were also calculated as 50% kernel_plug-in_.

We tested home range data for normality using the Shapiro-Wilk normality test, and plotted to identify outliers. Further, we tested correlation between home range and core area sizes (Spearman). Finally, seasonal differences in the size of home ranges and core areas were tested accounting for non-normality of the data and presence of outliers. Given the relatively small sample size available, both a Wilcoxon rank-sum test, which is known to have reduced power at small sample sizes, and a t-test for equal variances run on preliminary ranked data [44] were performed.

#### Influence of seasonality on the temporal dimension of movement: nightly activity patterns

We examined seasonal variation in activity by describing nightly activity patterns separately for dry and rainy season and subsequently testing the differences. We utilised minimum distance travelled (hereafter only “distance travelled”) per hour as a proxy for activity across each foraging night. This was measured as the linear distance between consecutive fixes 1 h apart, defined as “time intervals” (e.g. 18:00 to 19:00, 19:00 to 20:00, etc.), including all one-hour time intervals from 18:00 to 5:00. To ensure homogeneity of the data, measurements from consecutive fixes which where more than 1 h apart (occurring e.g. if fixes failed to record, leading to consecutive fixes being > 1 h apart) were discarded. Distance travelled as here defined is a measure that most likely underestimates actual distance travelled and activity. However, as it provides a standardised way of comparing movements at various moments in time, it represents a valuable tool for identifying activity patterns in space exploration, particularly in the case of a predator with hang-and-wait strategy.

We provide descriptive values of averages across individuals of distance travelled per hour and per night. Within each bat, these variables are measured, respectively, as the mean and sum of the values of distance travelled in each time interval, previously averaged across nights. Only individuals that presented at least one measurement per time interval were included in these calculations.

Nightly activity patterns within each season were analysed by testing for differences in distance travelled per hour across time intervals using linear mixed-effects models. To reduce skewness in the data, distance travelled was log-transformed prior to the analyses. Two models were run separately, one for each season (“within season” models) and built as follows: log-distance travelled per hour was modelled against time interval (categorical) as the explanatory variable and bat ID as a random factor to account for inter-individual variability. To test the overall significance of the time interval an Anova was run for each model. To obtain a pairwise comparison of means (Tukey contrasts) between time intervals we performed Post-Hoc analyses using the ‘multcomp’ package. Contribution of the random factor (ID) to explaining model variance was tested with a restricted likelihood ratio test (R package ‘RLRsim’).

We next tested for differences in patterns of distance travelled per hour between dry and rainy seasons using linear mixed-effects models (hereafter “between-seasons” models), where log-distance travelled per hour was modelled against season as the explanatory variable and bat ID as a random factor. For this analysis, a separate model was run for each time interval. The target of this analysis is to investigate the difference between the two seasons for a certain time interval. This test design, in contrast to a unique model including both season and time interval as explanatory variables, is therefore required to avoid the variance across time intervals within one season from obscuring the seasonal differences.

All data management and statistical analyses were performed in the R environment, version 3.4.4 [45] and significance was set at α = 0.05. *P*-values comprised between 0.05 and 0.1 are also reported.

#### Home range overlap

Estimates of home ranges and core areas were used to study spatial segregation among bats by determining the degree of overlap between neighbouring individuals. Any two bats were defined as neighbours when showing overlap in their 100% kernels_plug-in_ and subsequently identified as a dyad for the scope of the analysis. Using the 100% isopleth allowed us to include bats that, despite not having overlapping home ranges as defined in this study, still had the potential to encounter one another (for example, the dyad 14D-15D, Table 2). Furthermore, this method simultaneously excludes those dyads whose bats were unlikely to make contact during daily movements because they were either too far apart or were separated by other territories. The coefficient of overlap (CO) of a dyad was measured as follows: for both individuals of a dyad we calculated the ratio between overlap area and home range (or core area). The CO was then represented as the geometric mean of these two ratios. Maps of home ranges were created in Esri ArcMap, version 10.3.1 [46].

**Table 2:** Coefficients of overlap (CO) in the home range and core area of the dyads considered for the analyses of territoriality. Females are identified by the ‘f’ in the ID

Dyad type	Season	Dyad	Home range CO	Core area CO
male-male	Rainy	2R-3R	8.68	0.00
		4R-5R	3.07	0.00
		4R-6R	10.34	7.98
		8R-9R	0.02	0.00
	Dry	14D-15D	0.00	0.00
female-male	Rainy	1Rf-2R	86.64	58.61
		1Rf-3R	6.00	0.00
	Dry	14D-16Df	74.68	61.24
		15D-16Df	0.14	0.00
		17D-18Df	63.94	53.36
		17D-19Df	59.76	53.71
female-female	Dry	18Df-19Df	80.76	61.25

## Results

### Influence of seasonality on the spatial dimension of movement: home range

Home range size and core areas averaged, respectively, 5.46 ± 11.04 ha (range 0.45–42.32 ha, *n* = 22) and 0.74 ± 2.19 ha (range 0.02–10.7 ha, *n* = 22) (see Table 1 for individual values and Fig. 1b, c, d and Additional file 1: Figure S4 for visual representation of home ranges). Capture points of bats were located within their home ranges. We observed a positive correlation between home range and core area size (Spearman’s rho = 0.81, *P* < 0.001, *n* = 22).

Home range and core area values for dry and rainy seasons measured, respectively, 6.88 ± 11.66 ha (median 1.95 ha) and 0.39 ± 0.41 ha (median 0.24 ha, dry), and 4.47 ± 11.41 ha (median 1.3 ha) and 0.99 ± 2.92 ha (median 0.17 ha, rainy). The Wilcoxon rank-sum (W = 88, *p* = 0.051) and the t-test on ranks (t = 2.129, df = 20, *p* = 0.046) detected a marginally significant trend for the home ranges to be larger during the dry season compared to the rainy season. No significant seasonal difference in size was detected for the core areas (Wilcoxon rank-sum test, W = 75, df = 20, *p* = 0.29; t-test on ranks, t = 1.108, df = 20, *p* = 0.28).

### Influence of seasonality on the temporal dimension of movement: nightly activity patterns

**Fig. 2:**
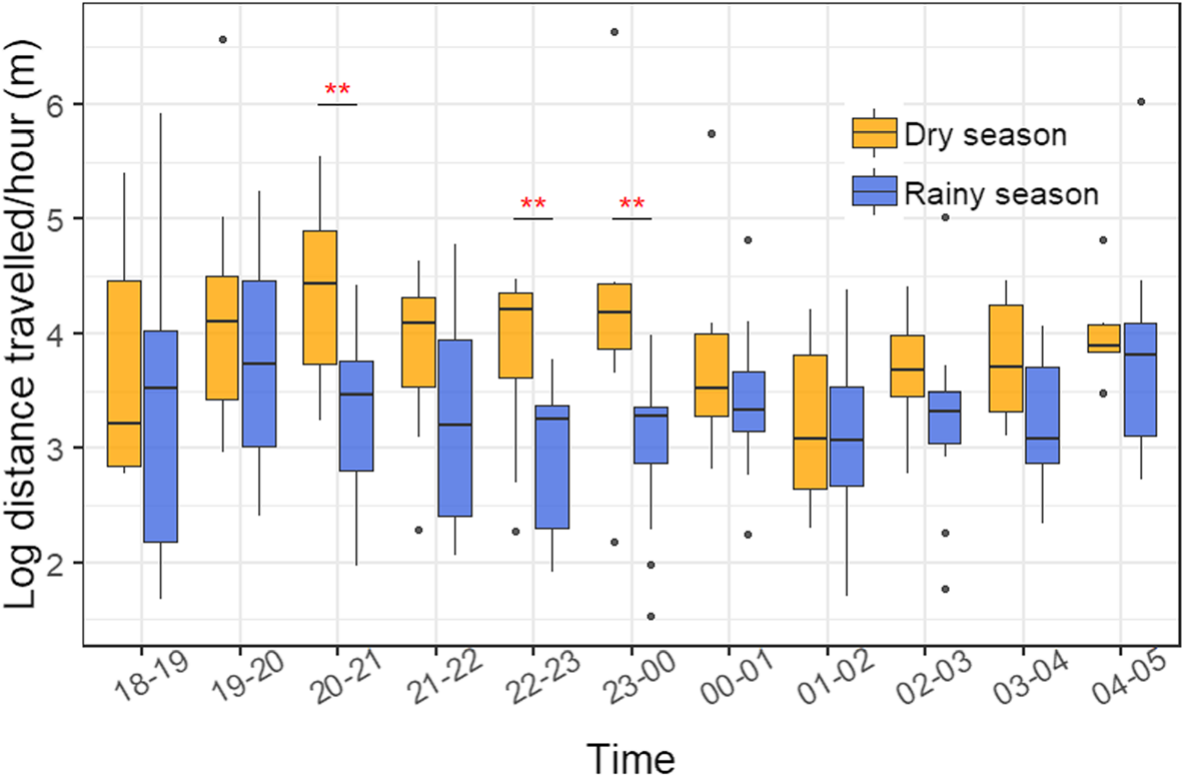
Variation in log-minimum distance travelled/hour across night and seasons. Each value represents the average log-minimum distance travelled/hour per bat at each time interval. Asterisks indicate significant differences between the two seasons for a certain time interval. See in Additional file 1: Figure S5 for patterns of distance travelled/hour at the individual level

Minimum distance travelled per night and per hour measured, respectively, 1100.20 ± 1356.22 m and 99.69 ± 123.42 m. Within-season models detected a significant difference in distance travelled per hour across the night during the dry (Anova, F = 2.39, *P* < 0.01) and the rainy (Anova, F = 2.74, *P* = 0.026) seasons (Fig. 2, Additional file 1: Figure S5). In particular, 20:00–21:00 and 23:00–00:00 appeared to be the periods with the greatest distance travelled in the dry season, while a low peak occurred at 01:00–02:00 (Tukey contrasts, *P* < 0.01 and *P* = 0.014, respectively). In the rainy season, distance travelled was highest early in the night (19:00–20:00) and just before sunrise (04:00–05:00), while the lowest peaks were located at 22:00–23:00, 23:00–00:00 and 01:00–02:00 (pairwise comparisons: 19:00–20:00 vs 22:00–23:00 (*p* = 0.051), 23:00–00:00 (*p* = 0.047), 01:00–02:00 (*P* = 0.09) and 04:00–05:00 vs 22:00–23:00 (*p* = 0.022), 23:00–00:00 (*p* = 0.02), 01:00–02:00 (*p* = 0.04), see Fig. 2). For both seasons, the individual ID included as a random factor significantly contributed to explaining model variance (dry: RLRT = 36.62, *P* < 0.001; rainy: RLRT = 67.46, *P* < 0.001).

Between-seasons models detected significantly greater distance travelled during the dry season compared to the rainy season for time intervals 20:00–21:00 (*P* < 0.01), 22:00–23:00 (*P* < 0.01) and 23:00–00:00 (*P* < 0.01), and non-significant differences at all other time intervals (Fig. 2).

### Home range overlap

A total of 12 dyads were identified. Of these, 6 were tracked during the dry season (1 male-male, 4 female-male, 1 female-female) and 6 during the rainy season (4 male-male, 2 female-male, see Table 2). Females exhibited large home range overlap (CO > 59%) and core area (CO > 53%) values with maximum one male (Table 2). Where two males and one female where tracked at the same tagging point in the same season (2 cases, Fig. 1b), females largely overlapped with one male, but not with the other (home range CO < 6%, core area CO = 0%, Table 2). During the dry season, two females were observed having high CO (home range CO = 80.76, core area CO = 61.25, Fig. 1d). Male-male dyads showed low or no overlap in both home ranges (CO < 11%) and core areas (CO < 8%) during dry and rainy seasons (Table 2, Fig. 1b-c).

## Discussion

### Influence of seasonality on the spatial and temporal dimensions of movement

Our results suggest changes in movement patterns both at the spatial and temporal dimensions, which are in line with our prediction that *L. frons* need to cover larger areas and be active for longer during the dry season to ensure enough resources are located.

In the area of study, insects are most abundant during the brief rainy seasons, while dry seasons are times of insect scarcity [22, 32]. The trend in showing larger home ranges during the dry season might be explained with the need for incorporating additional potential sources of prey when low abundances are prevalent. Patterns of seasonal changes in home range size linked to resource availability have been described for bats in temperate regions [47, 48]. In particular, Popa-Lisseanu, Bontadina [47] have hypothesised the role of insect scarcity linked to arid summer conditions in the home range enlargement of giant noctules *Nyctalus lasiupterus* in the Mediterranean region. Despite the challenges linked to our methodology, e.g. relatively low number of fixes/individuals and different individuals tracked in the two seasons, the data reveal the presence of weak effects providing fundamental insights into the ecology of *L. frons*.

While activity during the rainy season followed a bimodal pattern typical of aerial insectivores [49–51], showing minimum values in the middle of the night between 22:00 and 02:00, bats maintained high levels of activity for a prolonged time during the dry season and exhibited a decrease only between 01:00 and 02:00 (Fig. 2). These differences in nightly activity patterns between the two seasons further supported our hypothesis that *L. frons* may have to invest more to locate enough food during the dry season. During the dry season, *L. frons* has been reported to perch only briefly and move from tree to tree until a concentration of insects to exploit is found [28]. Additionally, recent studies show that bats relying on ephemeral resources, as it could be the case for *L. frons* during the dry season [28], tend to visit a greater number of foraging sites per night and exhibits greater variation in activity across nights compared to bats that rely on predicable resources [52, 53]. Similarly, farther displacements per hour in *L. frons* may be induced by moving along a larger number of trees until a profitable feeding site is located. Particularly in the dry season, the activity extension until later in the night may be needed to assure enough resources are secured. Conversely, during the rainy season previous reports have shown *L. frons* to reach satiation soon after emergence [28], thus matching the pattern presented here of early cessation of activity in this period. Furthermore, evening and morning peaks in activity could also be determined by territory patrolling behaviour [28]. A similar increase of extension of activity in dry seasons has been observed in other bat species in arid environments. For example, the other African Megadermatidae bat, *Cardioderma cor*, was found to increase foraging effort from the rainy to the dry season by elongating the activity time and foraging throughout the night [54].

The increase in movement detected in our study during the dry season can prove particularly costly, as the energy expenditure of flight in bats is estimated to be up to 15 times greater than basal metabolic rate [55] and may similarly severely increase water losses through evaporation [5]. It is further worth considering that the seasonal changes described here refer to the comparison between the long rainy (March to May) and short dry (January and February) seasons, and that results may be different, with potentially more pronounced differences, if considering the long dry (June to October) season. During the long dry season, due to the prolonged drought, the groundwater reservoir drops to the minimum, leading to the defoliation of most plants [56], including acacias, and a more severe drop in insect abundance is observed [57]. Such conditions, especially in a drought year, likely constitute a moment when survival of *L. frons* is most at risk and reflects on individual movements.

During fieldwork, we also observed that ephemeral wells dug by local communities were the only open water sources available to *L. frons* during the dry season. Given the high manoeuvrability of *L. frons*, we cannot exclude that the bats may opportunistically exploit the wells. However, this resource is discontinuous in the environment and temporary in nature, and the wells may be too narrow and deep to allow access during harsh seasons. Although desert-dwelling bats have been shown to converge around the rare waterbodies for water balance restoration and foraging [14, 19], we did not observe bat movement to reach the lake, which was in most cases located at a > 10 km distance from the sites where bats were captured, or their convergence on specific points of the landscape. We consider that, at least during dry seasons, *L. frons* may be independent from open water sources and rely exclusively on insects for fulfilling both water and energy requirements. Although independence from open water sources in deserts has been found for species in various taxa including mammals, e.g. rodents [58, 59], reports of this behaviour for bats in the wild are limited to field observations [21] (but see [60] for tests in captivity), and further investigations are needed.

### Home range overlap

In our study, we observed a clear trend for tagged males, or potential pairs, to maintain separate home ranges and core areas from those of other males in both the dry and rainy seasons. These findings are in line with previous evidence from observational studies that *L. frons* partitions the space into territories throughout the year [28]. Especially in the dry season, the need to patrol and actively defend a larger home range could also be associated with the identification of suitable foraging sites [28]. In only one case, two males (bats 4R and 6R, Fig. 1c), showed a larger degree of overlap than the other male-male dyads. The degree of territoriality in animals is known to vary with life stage (e.g. [61], and bat 4R could possibly be exhibiting a more explorative behaviour than others not showing a clearly defined territory, which is also reflected by its exceptionally large home range (42.32 ha, Table 1).

Females have been observed sharing home range with only one male, even in the presence of other neighbouring males, with which the overlap was restricted to the home range boundaries. These patterns of overlap further pointed to an organisation of *L. frons* in pairs or family units and potentially to the social monogamy of this species, which was previously assessed via behavioural observations [28, 62]. However, we recorded a male overlapping with two females. It is possible that these three bats constitute a pair with a fully-grown young. In fact, the period of parent-young association in *L. frons* was shown to be extended up to 3 months post-partum, likely to increase the chances of the young surviving the harsh dry season [62]. However, it should be noted that, given the design of this study, the account of home range overlap presented here is likely not a complete picture, as tracking was performed at different sites and not all individuals captured at a certain site were tracked.

## Conclusions and future directions

This study contributes to a better understanding of the still highly overlooked ecology of desert-dwelling bats. To cope with seasonally harsh climate conditions, *L. frons* appears to move over larger areas and to increase the duration of its nightly activity period, therefore suggesting greater efforts in prey search and a potentially lower cost-efficiency of the foraging activity. To explore the generalisation of these finding, we suggest that future studies would target different low-mobility species across a variety of deserts. Lastly, given the increasing aridity and habitat degradation that climate change is expected to determine in arid environments, we advocate attention being directed to the identification of aridity limits beyond which the presence of bats in these environments might be affected.

## Additional files

Additional file 1: **Figure S1.** River habitat during the dry season. **Figure S2.** River habitat during the rainy season. **Figure S3.** Skin of one tracked bat after manual tag removal. **Figure S4.** Locations and home ranges for tracked bats not presented in the main text (see Additional file 2 for details of home range estimation). **Figure S5.** Variation in log-minimum distance travelled/hour across individuals, night and seasons. (DOCX 3842 kb)

Additional file 2: Additional information on the selection of the home range estimator. (DOCX 14 kb)

## Data Availability

The data used in this study are available on the Movebank Data Repository: 10.5441/001/1.n157b4n8.

## Abbreviations

CO: Coefficient of Overlap
GPS: Global Positioning System
