# Supplementary material for: Movement seasonality in a desert-dwelling bat revealed by miniature GPS loggers
Source: Mov Ecol. 2019 Aug 16;7:27. doi: 10.1186/s40462-019-0170-8 (PMC6696681; doi:10.1186/s40462-019-0170-8)
Supplement: Supplementary file 1 — Figure S1. River habitat during the dry season. Figure S2. River habitat during the rainy season. Figure S3. Skin of one tracked bat after manual tag removal. Figure S4. Locations and home ranges for tracked bats not presented in the main text (see Additional file 2 for details of home range estimation). Figure S5. Variation in log-minimum distance travelled/hour across individuals, night and seasons. (DOCX 3842 kb) [file 40462_2019_170_MOESM1_ESM.docx]

**Additional File 1**

**Movement seasonality in a desert-dwelling bat revealed by miniature GPS loggers**

Irene Conenna*, Adrià López-Baucells, Ricardo Rocha, Simon Ripperger & Mar Cabeza

*^*^ Corresponding author:* Irene Conenna*; E-mail:* [*irene.conenna@gmail.com*](mailto:irene.conenna@gmail.com)

**This supplementary material contains:**

**Figure S1.** River habitat during the dry season.

**Figure S2.** River habitat during the rainy season.

**Figure S3.** Skin of one tracked bat after manual tag removal.

**Figure S4.** Locations and home ranges for tracked bats not presented in the main text (see Additional File 2 for details of home range estimation).

**Figure S5.** Variation in log-minimum distance travelled/hour across individuals, night and seasons.

Figure S1. Example of river habitat during the dry season (credits: Joni Uusitalo).


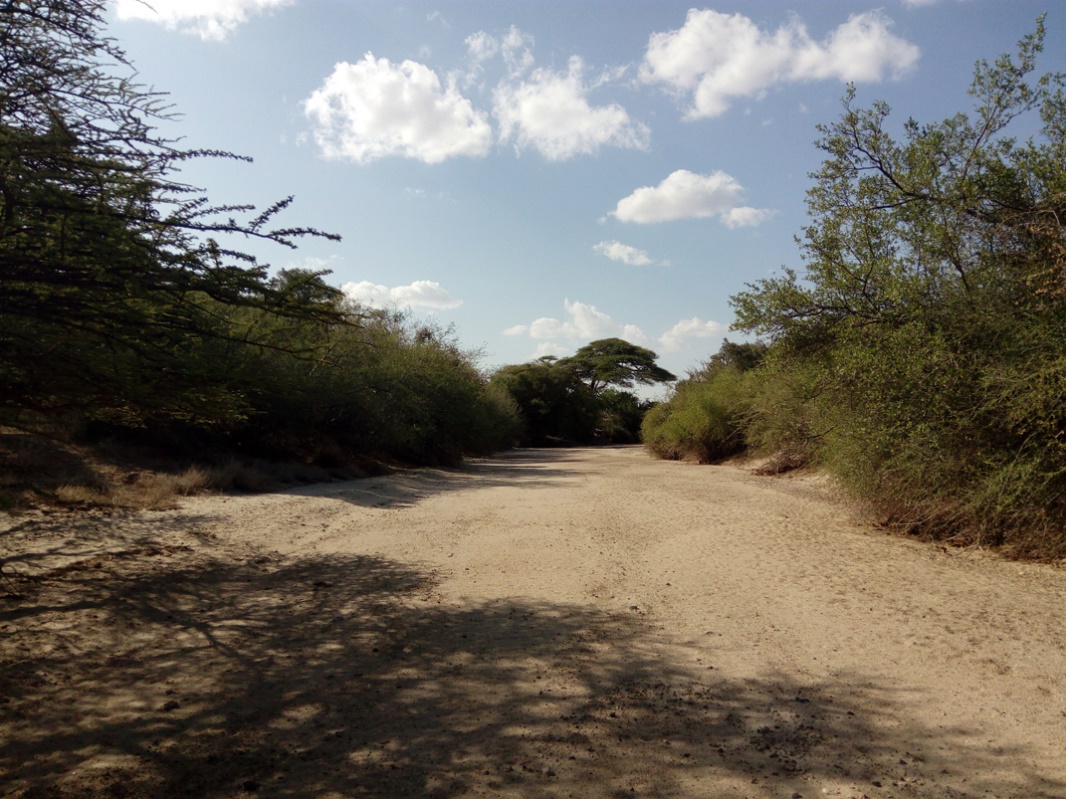


Figure S2. Example of river habitat after flooding during the rainy season (credits: Miquel Torrents-Ticó).


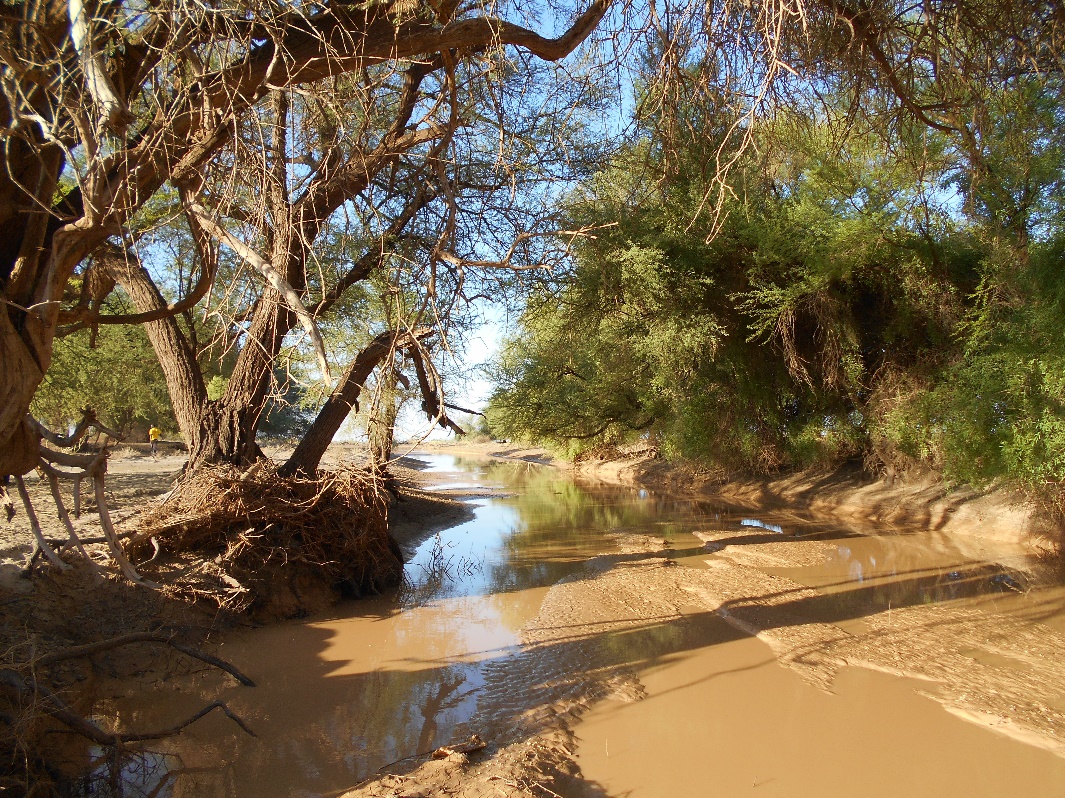


Figure S3. Condition of the skin of one of the *Lavia frons* tracked after manual tag removal (credits: Irene Conenna).


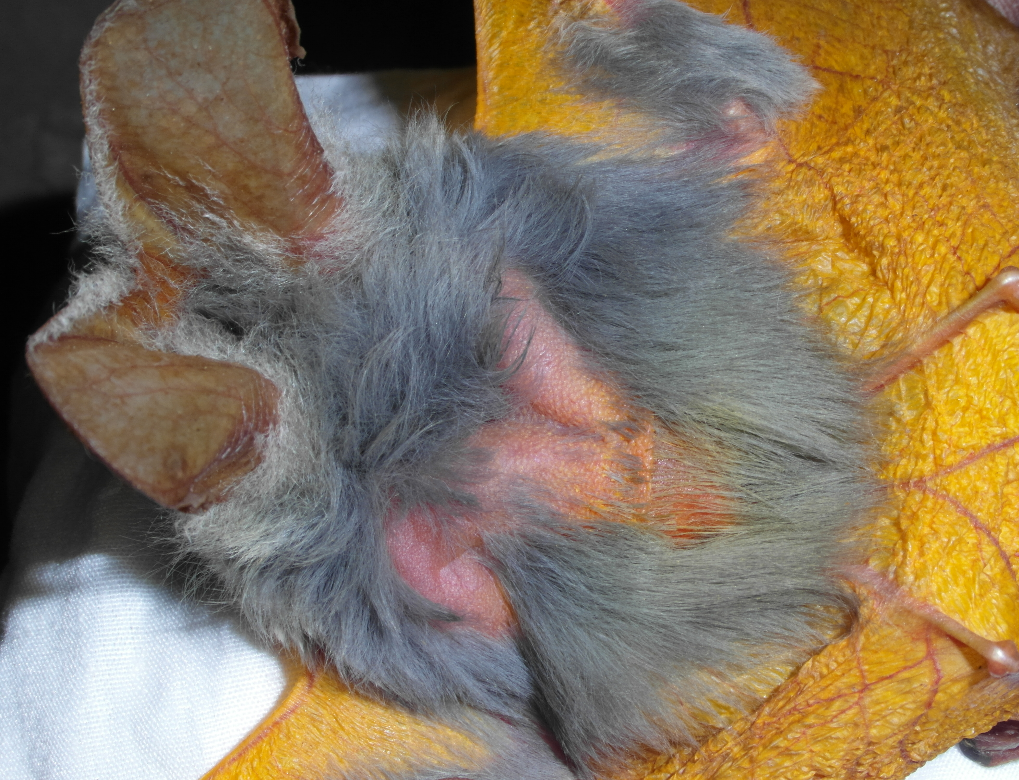


Figure S4. Panel A to F show recorded locations (dots) and respective home range boundaries (lines) for individuals tracked in tagging point (TP) 3 (A), TP4 (B and C) during the rainy season, and TP1 (D), TP3 (E) and TP4 (F) during the dry season.


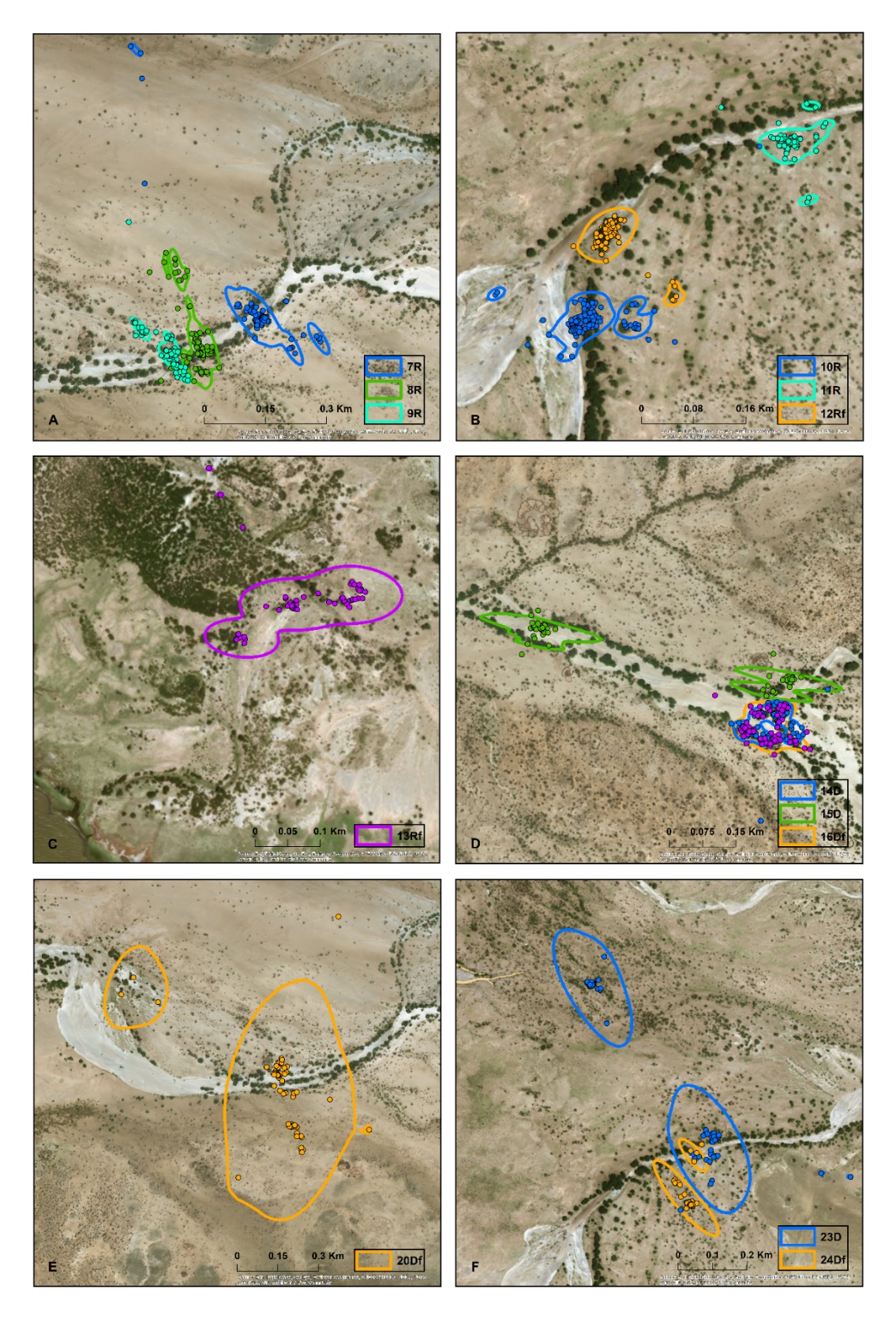


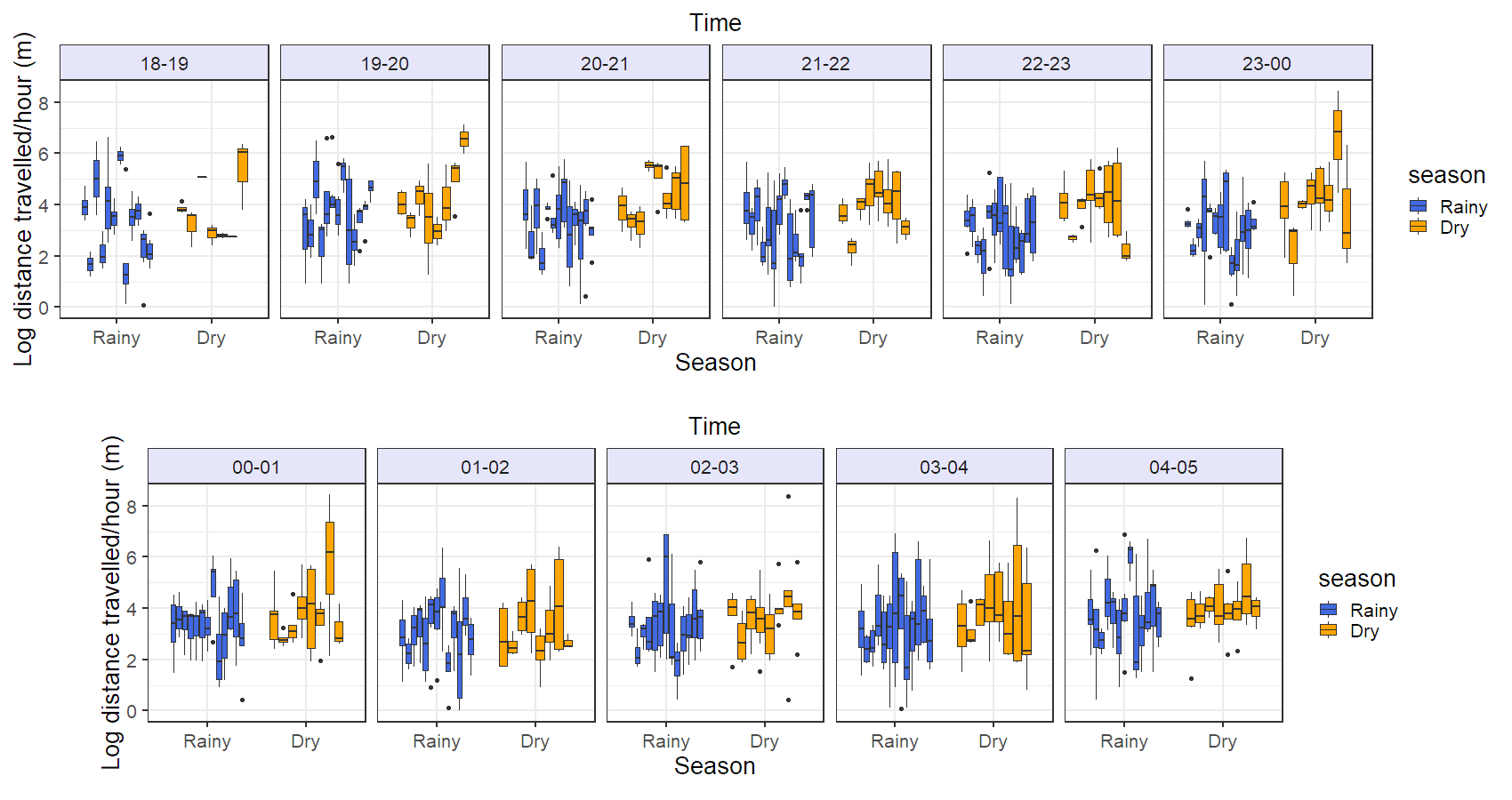
Figure S5. Variation in log-minimum distance travelled/hour across individuals, night and seasons. Each data point represents raw values of log-minimum distance travelled/hour per bat at each time interval.
