# Supplementary material for: Movement seasonality in a desert-dwelling bat revealed by miniature GPS loggers
Source: Mov Ecol. 2019 Aug 16;7:27. doi: 10.1186/s40462-019-0170-8 (PMC6696681; doi:10.1186/s40462-019-0170-8)
Supplement: Supplementary file 2 — Additional information on the selection of the home range estimator. (DOCX 14 kb) [file 40462_2019_170_MOESM2_ESM.docx]

**Additional File 2**

**Movement seasonality in a desert-dwelling bat revealed by miniature GPS loggers**

Irene Conenna*, Adrià López-Baucells, Ricardo Rocha, Simon Ripperger & Mar Cabeza

*^*^ Corresponding author:* Irene Conenna*; E-mail:* [*irene.conenna@gmail.com*](mailto:irene.conenna@gmail.com)

**This supplementary material contains:**

**Home range estimation**

**Home range estimation**

When estimating home ranges, the same estimator can perform well or poorly depending on the characteristic of the data under investigation (1-5). The factors that are known to affect home range estimation, in addition to the temporal autocorrelation discussed in the main text, are samples size, distribution shape of locations (determined by species ecology) and landscape features (e.g., barriers). Based on these factors, we explain in detail the reasons for the selection of the kernel_plug-in_ estimator for the data under study, and in particular a) the choice of the Kernel Density Estimation (KDE) over other major estimators, and b) the choice of the plug-in as method to calculate the smoothing factor for the KDE.

a) KDE constitutes a non-parametric method to calculate utilisation distributions (UD), i.e. probabilistic models of the use of space by the animal, from which home ranges can be measured with different confidence levels (100%, 95%, 50%, etc.) (6). KDE was selected since it is considered to produce less biased estimates and to be less sensitive to sample size and outliers than the traditional Minimum Convex Polygon (1, 7), and because it is found to produce a better fit than the more recent Local Convex Hull methods when no barriers or hard boundaries are present in the home ranges (3, 4). No barriers that could constrain movement were present in our study area and, despite likely in different proportions (not analysed in this study), *L. frons* appeared to move over both dry rivers and bushland matrix.

b) In KDE, the final shape of the utilisation distribution is smoothened by a tuning parameter, the bandwidth h or smoothing factor, for which estimation various methods have been developed. This makes KDE both flexible and sensitive to the choice of the method used to estimate the bandwidth h. For our analysis, the plug-in method was selected since it was shown to outperform others in estimating the utilisation distribution for sample sizes comparable to the ones in this study, and for the distributions observed for our data, i.e. either clustering of points around the roosting site or a multimodal pattern connected by corridors (4). Other commonly employed methods to calculate the bandwidth h are the Reference and the Least Squares Cross Validation (8). These were excluded because not suitable to fit multimodal distributions and data with high level of clusters or duplicated points, respectively (1).

**References**

1. Gitzen RA, Millspaugh JJ, Kernohan BJ. Bandwidth selection for fixed‐kernel analysis of animal utilization distributions. The Journal of Wildlife Management. 2006;70(5):1334-44.

2. Seaman DE, Millspaugh JJ, Kernohan BJ, Brundige GC, Raedeke KJ, Gitzen RA. Effects of sample size on kernel home range estimates. The Journal of Wildlife Management. 1999:739-47.

3. Getz WM, Wilmers CC. A local nearest‐neighbor convex‐hull construction of home ranges and utilization distributions. Ecography. 2004;27(4):489-505.

4. Lichti NI, Swihart RK. Estimating utilization distributions with kernel versus local convex hull methods. The Journal of Wildlife Management. 2011;75(2):413-22.

5. Börger L, Franconi N, De Michele G, Gantz A, Meschi F, Manica A, et al. Effects of sampling regime on the mean and variance of home range size estimates. Journal of Animal Ecology. 2006;75(6):1393-405.

6. Worton BJ. Kernel methods for estimating the utilization distribution in home‐range studies. Ecology. 1989;70(1):164-8.

7. White GC, Garrott RA. Analysis of wildlife radio-tracking data: Elsevier; 2012.

8. Hemson G, Johnson P, South A, Kenward R, Ripley R, MACDONALD D. Are kernels the mustard? Data from global positioning system (GPS) collars suggests problems for kernel home‐range analyses with least‐squares cross‐validation. Journal of Animal Ecology. 2005;74(3):455-63.
